# Clinical utility of cardiovascular magnetic resonance imaging in patients with implantable cardioverter defibrillators presenting with electrical instability or worsening heart failure symptoms

**DOI:** 10.1186/s12968-020-00609-z

**Published:** 2020-05-11

**Authors:** Frank Lindemann, Sabrina Oebel, Ingo Paetsch, Arash Arya, Nikolaos Dagres, Sergio Richter, Borislav Dinov, Sebastian Hilbert, Susanne Loebe, Clara Stegmann, Michael Doering, Andreas Bollmann, Gerhard Hindricks, Cosima Jahnke

**Affiliations:** Department of Electrophysiology, Heart Center Leipzig at University of Leipzig, Struempellstr. 39, 04289 Leipzig, Germany

**Keywords:** Cardiovascular magnetic resonance imaging, Implanted cardioverter defibrillator, Diagnostic reclassification, Ventricular tachycardia ablation, Treatment decision

## Abstract

**Background:**

Data on the usefulness of cardiovascular magnetic resonance (CMR) imaging for clinical decision making in patients with an implanted cardioverter defibrillator (ICD) are scarce. The present study determined the impact of CMR imaging on diagnostic stratification and treatment decisions in ICD patients presenting with electrical instability or progressive heart failure symptoms.

**Methods:**

212 consecutive ICD patients underwent 1.5 T CMR combining diagnostic imaging modules tailored to the individual clinical indication (ventricular function assessment, myocardial tissue characterization, adenosine stress-perfusion, 3D-contrast-enhanced angiography); four CMR examinations (4/212, 2%) were excluded due to non-diagnostic CMR image quality. The resultant change in diagnosis or clinical management was determined in the overall population and compared between ICD patients for primary (115/208, 55%) or secondary prevention (93/208, 45%). Referral indication consisted of documented ventricular tachycardia, inadequate device therapy or progressive heart failure symptoms.

**Results:**

Overall, CMR imaging data changed diagnosis in 40% (83/208) with a significant difference between primary versus secondary prevention ICD patients (37/115, 32% versus 46/93, 49%, respectively; *p* = 0.01). The information gain from CMR led to an overall change in treatment in 21% (43/208) with a similar distribution in primary versus secondary prevention ICD patients (25/115,22% versus 18/93,19%, *p* = 0.67). The effect on treatment change was highest in patients initially scheduled for ventricular tachycardia ablation procedure (18/141, 13%) with revision of the treatment plan to medical therapy or coronary revascularization.

**Conclusions:**

CMR imaging in ICD patients presenting with electrical instability or worsening heart failure symptoms provided diagnostic or management-changing information in a considerable proportion (40% and 21%, respectively).

## Background

Over the past decade, increasing evidence has been accumulated regarding the safety of magnetic resonance (MR) imaging in patients with cardiovascular implantable electronic devices [1–3]. In general, MR scanning has been found to be safe when conducted under careful supervision in both MR conditional and conventional active rhythm devices [4]. However, more recent research addressed the pivotal issue of achieving adequately high diagnostic image quality of cardiovascular magnetic resonance (CMR) imaging in patients with implanted cardioverter defibrillator (ICD) and utilized well-established spoiled-gradient echo cine sequences following gadolinium-containing contrast agent application in combination with wideband late-gadolinium enhancement (LGE) imaging. Thus, the fundamental diagnostic CMR modules of basic ventricular functional assessment and myocardial tissue characterization have eventually become available for routine clinical usage in ICD patients either [5–8]. While previous investigations have largely focused on safety and image quality, the clinical impact and usefulness of CMR imaging in ICD patients have not been evaluated yet. Hence, the current study investigated the impact of CMR imaging on diagnostic stratification and treatment decisions in ICD patients presenting with electrical instability or progressive heart failure symptoms.

## Methods

### Patient population

The study was conducted in accordance with the local institutional review board and the standards of the University of Leipzig ethics committee. Written informed consent was obtained from all patients prior to CMR examination. All patients with an ICD referred for clinical management of electrical instability and/or worsening heart failure were seen by the cardiologist/electrophysiologist in charge and the decision to undergo CMR imaging was at the discretion of the clinician. Consecutive patients referred for CMR imaging with an ICD between June 2015 and December 2018 formed the study population. The CMR examination rigorously followed a previously published, standardized protocol [5]. All patients underwent chest x–ray prior to the CMR examination and in the presence of abandoned, epicardial or fractured leads, CMR imaging was not performed (i.e. patients were not considered for study inclusion). In addition, the time period between device implantation and CMR examination was > 6 weeks.

### Device interrogation and programming

Directly prior to the CMR examination, an experienced electrophysiologist carried out a thorough ICD device interrogation in the scanner console room : battery status and sensing/pacing thresholds of all leads were documented and the device memory was evaluated for events (e.g. appropriately or inappropriately classified arrhythmias; date, number and kind of delivered antitachycardia pacing therapies etc.). Subsequently, devices were programmed for the CMR scanner environment: all tachyarrhythmia functions were switched off; MR–conditional labeled devices were programmed into the manufacturer–provided MR–safe mode settings; non–MR–conditional devices were programmed to pacing off, sensing–only (ODO or OVO) or to asynchronous pacing (VOO) depending on the intrinsic rhythm of the patient. The electrophysiologist and the programming device were present in the scanner console room throughout the entire CMR examination. Directly after completion of the CMR examination, patients underwent a repeat ICD interrogation in the console room and all devices were restored to their original settings.

### CMR imaging protocol

A cardiologist with a high level of expertise in CMR imaging (I.P. or C.J., both > 20 years of experience and Society for Cardiovascular Magnetic Resonance and European Society of Cardiology level 3 trained) was present throughout the entire procedure for online evaluation of all CMR images and to guide or adapt a previously established CMR workflow/imaging strategy for device patients if needed to fully address the clinical indication [5]. Briefly, CMR imaging protocols were tailored to the clinical indication and diagnostic imaging modules were combined accordingly (i.e. ventricular function assessment, myocardial tissue characterization using wideband LGE imaging plus/minus T1- or T2-weighted blackblood turbo spin echo sequences, myocardial perfusion assessment using dynamic first-pass perfusion spoiled-gradient echo imaging during adenosine stress and conventional contrast-enhanced three-dimensional angiography scans). Cine imaging was performed using spoiled gradient echo (SGE) sequences with short–axis geometries being acquired before and long–axis geometries after contrast agent application. For LGE imaging, the wideband technique was employed (bandwidth of the inversion prepulse, 3.000 Hz; frequency offset, + 1.000 Hz) [6]. Both, cine and LGE imaging sequences were acquired during end-expiratory breath-holding. All CMR examinations were carried out on a 1.5 T CMR scanner (Ingenia, Philips Healthcare, Best, The Netherlands) using a 28–element array coil with full in–coil signal digitalization and optical transmission. Following current recommendations, whole–body specific absorption rate (SAR) was restricted to 2 watts per kilogram bodyweight. Patients were continuously monitored throughout the procedure based on vector–surface electrocardiogram (ECG), peripheral pulse oximetry, respiratory motion pattern, non–invasive blood pressure measurements as well as continuous visual and voice contact. If online evaluation of initial short–axis cine images demonstrated ≥12 non–evaluable left–ventricular (LV) segments due to severe generator–related artifacts, the CMR examination was considered non–diagnostic and terminated.

### CMR image quality

Quality of CMR images (i.e. cine, LGE, T1- and T2-weighted blackblood turbo-spin echo, dynamic perfusion and contrast-enhanced three-dimensional angiographic image sequences) was evaluated offline > 6 weeks later by two experienced CMR imaging experts (I.P., C.J.) in a consensus read; device–related image artifacts were assessed per myocardial segment of the LV and right ventricle (RV) following previously defined classification systems [9, 10]. Myocardial segments were considered evaluable if artifact–free in at least one standard cardiac geometry.

### CMR–based change in diagnosis or treatment

In all patients, clinical data were recorded at the time of the CMR examination and the indication for CMR referral was documented: electrical instability was defined as electrical storm (i.e. ≥ 3 separate arrhythmia episodes leading to adequate ICD therapies consisting of anti-tachycardia pacing (ATP) or shocks occurring within a 24-h period), ≤ 2 adequate ATP/shock(s), or sustained ventricular tachycardia (VT) below detection; progressive heart failure was defined as any increase in New York Heart Association stage of at least one functional class during the last three months. The CMR imaging results were determined by the consensus of two experienced CMR imaging experts (I.P., C.J.) within < 1 h following the CMR examination and the CMR–based diagnosis was documented. CMR–based change in diagnosis was defined as a new, previously not considered diagnosis (i.e. by clinical history, other cardiac imaging modalities etc.). CMR–based change in treatment decision constituted the sole responsibility of the electrophysiologist/cardiologist in charge and was defined as change in management consisting of any deviation from the referral to invasive procedures (e.g. electrophysiology study/ablation procedure, cardiac surgery, coronary revascularization etc.) or a change in medical therapy.

### Statistical analysis

All analyses were done using SPSS (version 21, International Business Machines, Inc., Armonk, New York, USA). Continuous variables were given as mean ± standard deviation for normally distributed data; frequencies and percentages were used to describe categorical data. Differences between continuous and categorical variables were assessed using Student’s t–test and Chi–square test as appropriate. All tests were two–tailed and a p–value of < 0.05 was considered significant.

## Results

### Patient characteristics

CMR examinations were carried out in 212 consecutive ICD patients; for patients who underwent repeat/follow-up examinations, only the first CMR dataset was considered for analysis. Four CMR examinations (4/212, 2%) were excluded due to non-diagnostic CMR image quality. Hence, the final dataset for analysis comprised of 208 CMR examinations of ICD patients. Data analysis was conducted for all patients and for the subgroups of primary versus secondary prevention ICD patients (*n* = 115 versus *n* = 93, respectively). Overall, 94 patients were equipped with an MR-conditional device (94/208, 45%; see Table 1); patients with MR-conditional devices were younger (58 ± 12 versus 61 ± 12 years; *p* = 0.023) and had a shorter mean time since ICD implantation (48 ± 43 versus 68 ± 54 months; *p* = 0.003). Referral indication consisted of documented VT (141/208, 68%), inadequate device therapy (15/208, 7%) or progressive heart failure symptoms; detailed patient characteristics are given in Table 1. In all ICD patients, device interrogation before and after the CMR examination yielded no significant changes (mean change of battery status 0.0 ± 0.1 V; lead threshold, 0.0 ± 0.3 V; lead impedance, − 7.5 ± 52.7 Ω; for all p = ns).

**Table 1:** Patient Characteristics

	All patients (*n* = 208)	Primary prevention (*n* = 115)	Secondary prevention (*n* = 93)	p–value
Age, years	60 ± 12	60 ± 11	59 ± 13	0.532
Gender, male, n(%)	174 (84)	98 (85)	76 (82)	0.498
BMI, kg/m^2^	28.6 ± 5.1	29.2 ± 5.3	27.9 ± 4.8	0.062
Hypertension, n(%)	144 (69)	80 (70)	64 (69)	0.907
Diabetes, n(%)	46 (22)	32 (28)	14 (15)	0.027
CHA_2_DS_2_–VASc Score	2.7 ± 1.4	2.8 ± 1.2	2.4 ± 1.5	0.006
NYHA class	1.9 ± 0.9	2.1 ± 0.9	1.6 ± 1.0	0.001
Known CAD, n(%)	83 (40)	52 (45)	31 (33)	0.082
Severity of CAD				0.178
Single–vessel, n(%)	31 (37)	18 (35)	13 (42)	
Multi–vessel, n(%)	52 (63)	34 (65)	18 (58)	
Electrical instability				0.009
≤ 2 adequate ATP/shock(s), n(%)	23 (11)	16 (14)	7 (8)	
Electrical storm, n(%)	107 (51)	53 (46)	54 (58)	
Below detection, n(%)	11 (5)	3 (3)	8 (9)	
Inadequate ATP/shock(s), n(%)	15 (7)	6 (5)	9 (10)	
Prior VT ablation, n(%)	50 (24)	16 (14)	34 (37)	< 0.001
Antiarrhythmic drugs				
Betablockers, n(%)	189 (91)	104 (90)	85 (91)	0.811
Class I, n(%)	14 (7)	5 (4)	9 (10)	0.127
Class III, n(%)	60 (29)	31 (27)	29 (31)	0.504
Device characteristics				0.070
S–ICD, n(%)	6 (3)	5 (4)	1 (1)	
Single chamber ICD, n(%)	101 (49)	55 (48)	46 (49)	
Dual chamber ICD, n(%)	65 (31)	30 (26)	35 (38)	
CRT–D, n(%)	36 (17)	25 (22)	11 (12)	
MR-conditional devices, n(%)	94 (45)	48 (42)	46 (49)	0.266
Time since ICD implantation, months	58.9 ± 50.0	56.4 ± 42.4	61.9 ± 58.2	0.436
Range, months	1.4–240.4	1.6–225.0	1.4–240.4	

Values are mean ± SD or n (%); *p*-values are given for the comparison of primary versus secondary prevention. *BMI* body mass index; *CAD* coronary artery disease; *ATP* antitachycardia pacing; *VT* ventricular tachycardia; *ICD* implanted cardioverter defibrillator; *S–ICD* subcutaneous implantable cardioverter defibrillator; *CRT–D* cardiac resynchronization therapy defibrillator, *MR* magnetic resonance

### CMR imaging protocol

The following CMR diagnostic imaging modules were employed: cine imaging for ventricular function assessment (*n* = 208), myocardial tissue characterization (LGE, *n* = 206 + / - T1– or T2–weighted blackblood spin echo imaging, *n* = 49 or *n* = 55, respectively), resting or adenosine stress perfusion (*n* = 27) and contrast–enhanced 3D–angiography (pulmonary veins, *n* = 25 or thoracic aorta, *n* = 3). Results of CMR data are given in Table 2.

**Table 2:** CMR Examination Data

	All patients (*n* = 208)	Primary prevention (*n* = 115)	Secondary prevention (*n* = 93)	p–value
Heart rate, bpm	68 ± 14	70 ± 14	64 ± 13	0.002
Systolic BP, mmHg	127 ± 20	124 ± 20	130 ± 21	0.010
Diastolic BP, mmHg	73 ± 12	73 ± 12	74 ± 12	0.888
LVEDV, ml	249 ± 106	283 ± 111	207 ± 83	< 0.001
LVESV, ml	170 ± 102	207 ± 104	125 ± 78	< 0.001
LVEF, %	36 ± 15	30 ± 13	44 ± 13	< 0.001
RVEDV, ml	128 ± 65	133 ± 71	121 ± 55	0.163
RVESV, ml	62 ± 48	66 ± 53	57 ± 40	0.139
RVEF, %	54 ± 11	54 ± 12	56 ± 10	0.186
LGE positive (n = 206), n(%)	135 (66)	73 (65)	62 (67)	0.756
LV, LGE positive, n(%)	132 (64)	73 (65)	59 (63)	0.863
LV-LGE pattern (*n* = 132)				0.862
subendocardial, n(%)	82 (62)	47 (64)	35 (59)	
subepicardial, n(%)	14 (11)	7 (10)	7 (12)	
midwall striae, n(%)	23 (17)	13 (18)	10 (17)	
midwall patchy, n(%)	13 (10)	6 (8)	7 (12)	
LV-LGE, segments	4.1 ± 2.7	4.2 ± 2.8	4.0 ± 2.5	0.647
LV-LGE, % transmurality	73 ± 25	75 ± 24	70 ± 26	0.243
RV, LGE positive, n(%)	9 (4)	2 (2)	7 (8)	0.044
LV thrombus, n(%)	9 (4)	5 (4)	4 (4)	0.987

Values are mean ± SD or n (%); p-values are given for the comparison of primary versus secondary prevention. *BP* blood pressure; *LVEDV* left ventricular end–diastolic volume; *LVESV* left ventricular end–systolic volume; *LVEF* left ventricular ejection fraction; *LGE* late gadolinium enhancement; *RV* right ventricle;* LV LGE* pattern was categorized according to reference [11]

### CMR image quality

**Fig. 1:**
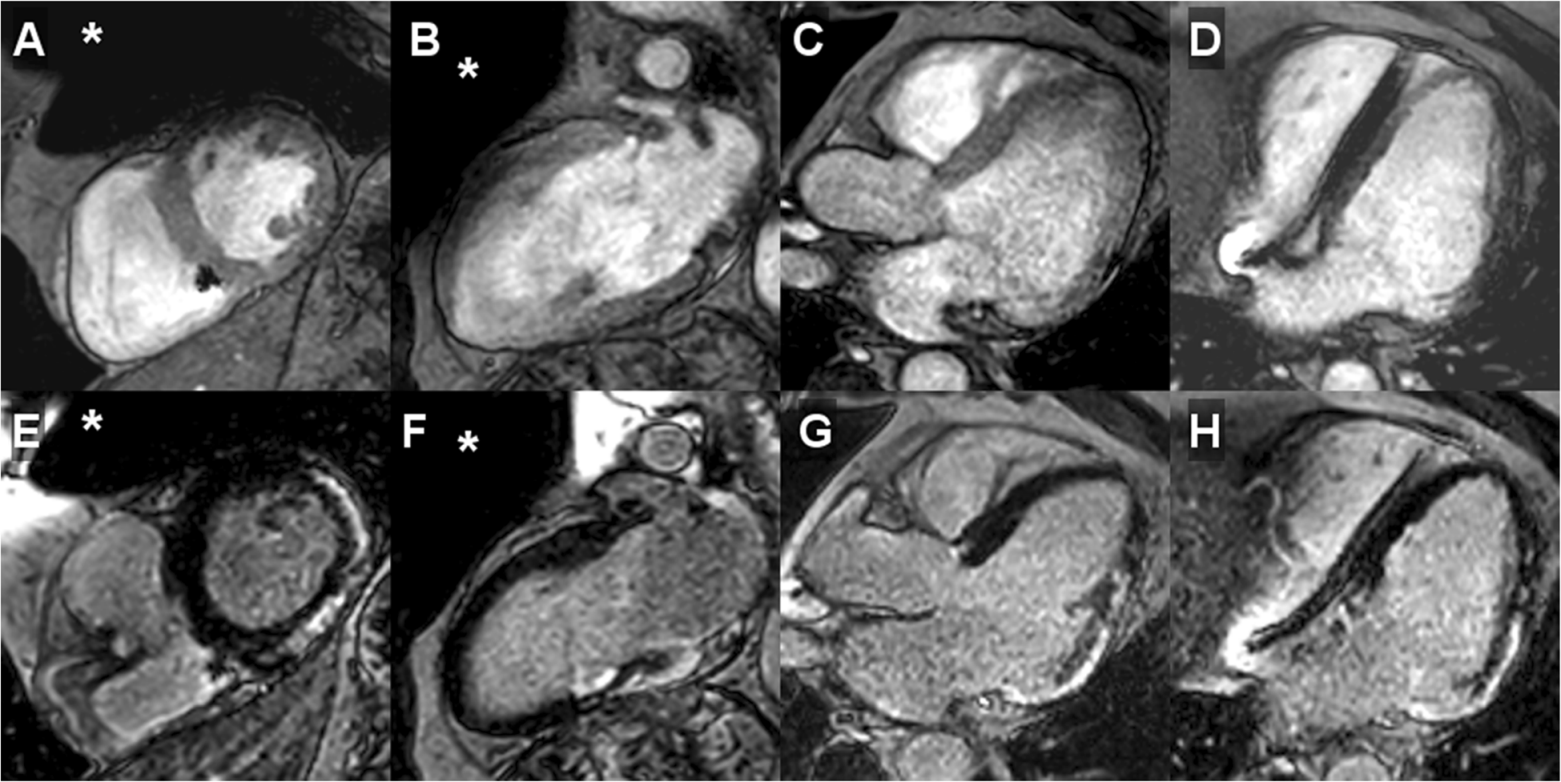
CMR imaging in a single-chamber, secondary prevention implanted cardioverter defibrillator (ICD) patient (male, 59 years) presenting with electrical storm (15 adequate episodes of antitachycardia pacing) 24.7 months after device implantation. Diagnostic change (+); management/treatment change (−): pre-CMR referral diagnosis: unknown cardiac disease; post-CMR diagnosis: post-infectious state of chronic myocarditis; pre-CMR treatment plan: ventricular tachycardia ablation; post-CMR treatment plan: targeted ventricular ablation procedure guided by late gadolinium enhancement (LGE)-defined ventricular substrate. A – D, post–contrast cine imaging in all cardiac standard geometries demonstrated regional wall motion abnormality (akinesis of basal inferolateral segment) with near-normal global systolic left-ventricular function (LVEF 52%); E – H, LGE CMR imaging in identical geometries revealed the presence of extensive, strictly epicardial regions of fibrosis. Approximate generator position is indicated by the asterisk

**Fig. 2:**
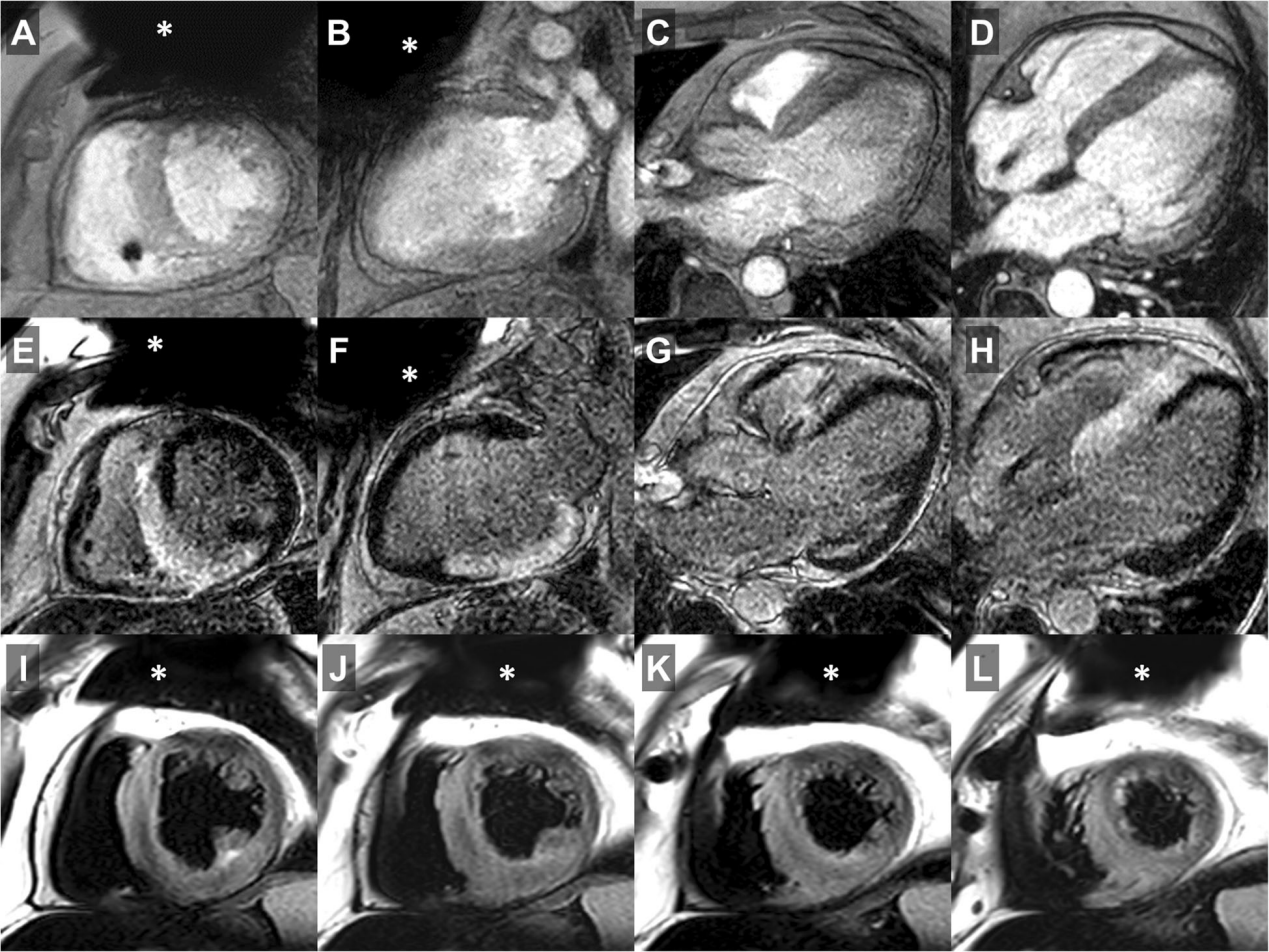
CMR imaging in a dual-chamber, secondary prevention ICD patient (male, 59 years) presenting with electrical storm (13 adequate shock episodes) 7.2 months after device implantation. Diagnostic change (+); management/treatment change (+): pre-CMR referral diagnosis: hypertensive heart disease; post-CMR diagnosis: acute disease state of cardiac sarcoidosis; pre-CMR treatment plan: ventricular tachycardia ablation; post-CMR treatment plan: medical therapy including steroids. A – D, post–contrast cine imaging in all cardiac standard geometries showed concentric left ventricular (LV) hypertrophy with mildly reduced global LV ejection fraction (LVEF 47%); E – H, LGE CMR imaging (identical geometries) identified extensive, mostly transmural areas of positive LGE in all septal and inferior LV segments with corresponding edema on T2-weighted turbo spin echo imaging (I – L, consecutive short-axis geometries) consistent with an acute inflammatory disease state of cardiac sarcoidosis subsequently being confirmed by endomyocardial biopsy. Approximate generator position is indicated by the asterisk

**Fig. 3:**
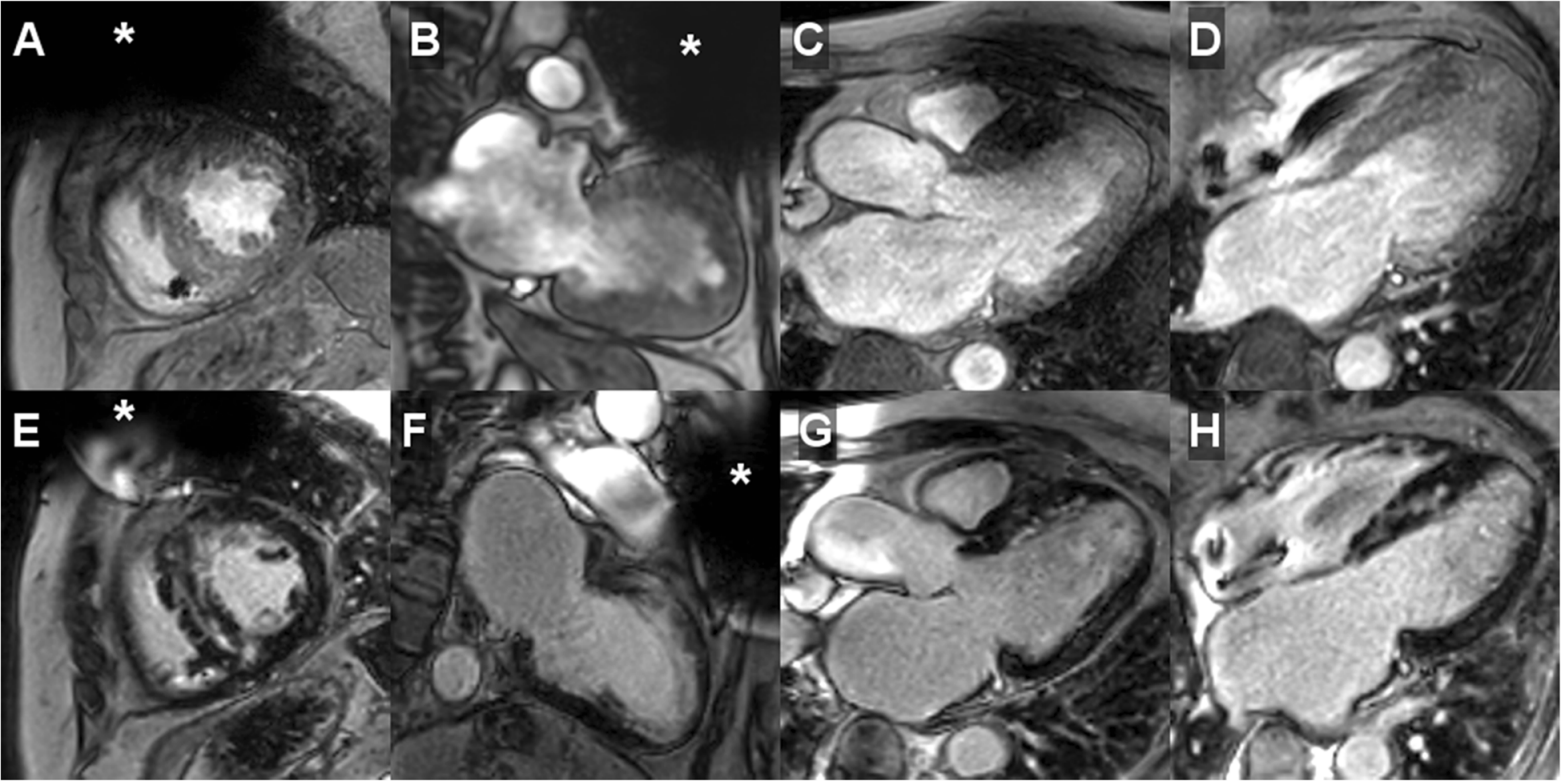
CMR imaging in a dual-chamber, secondary prevention ICD patient (female, 58 years) presenting with worsening heart failure symptoms 27.9 months after device implantation. Diagnostic change (+); management/treatment change (–): pre-CMR referral diagnosis: suspected myocardial storage disease; post-CMR diagnosis: hypertrophic cardiomyopathy; pre-CMR treatment plan: medical therapy; post-CMR treatment plan: medical therapy. A – D, on post–contrast cine imaging (cardiac standard geometries) LV septal hypertrophy was identified with normal regional wall motion/normal global systolic LV function (LVEF 59%); E – H, LGE CMR imaging demarcated the typical pattern of multifocal islands/patchy fibrosis in the interventricular septum while the remaining LV myocardium was normal. Approximate generator position is indicated by the asterisk

**Fig. 4:**
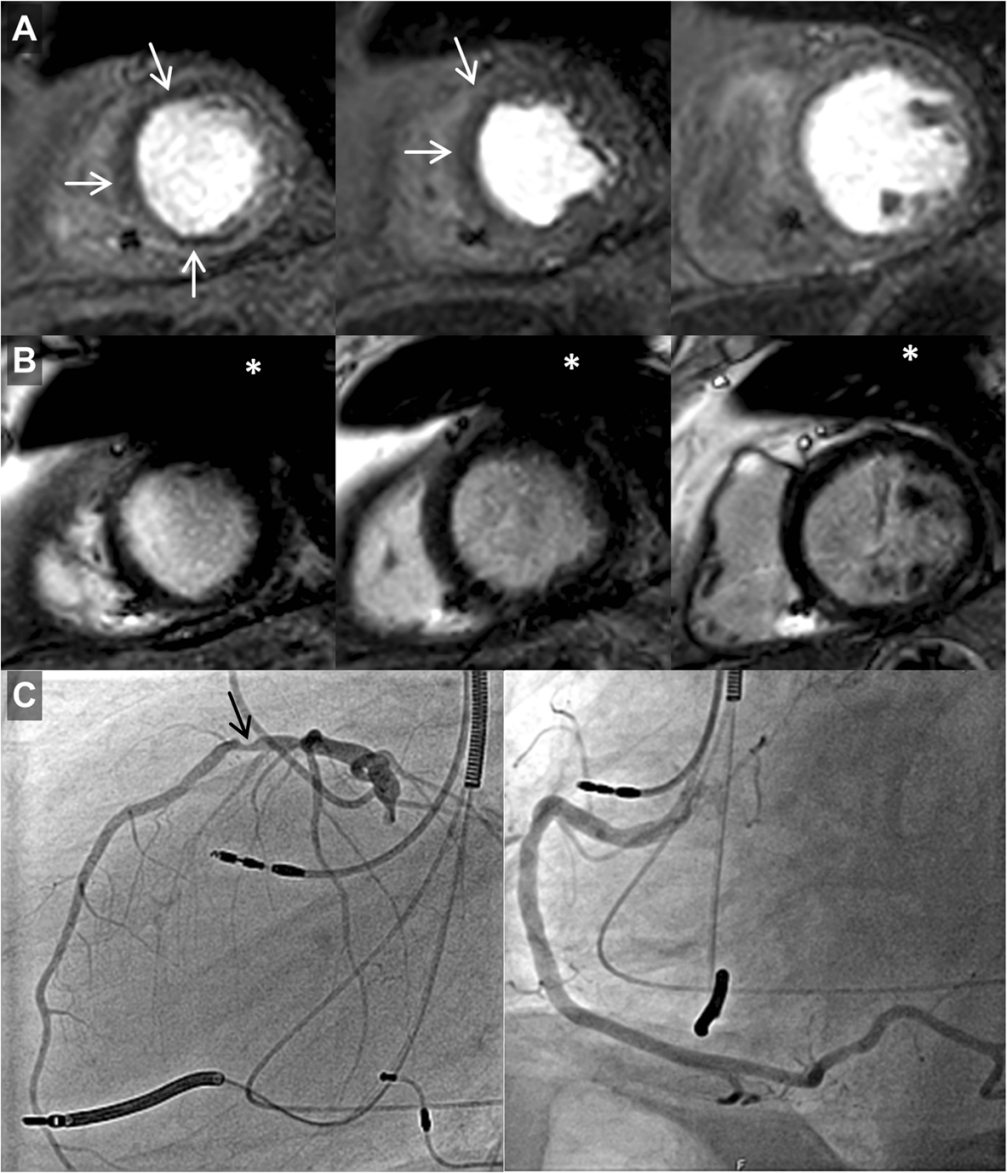
CMR imaging in a primary prevention ICD patient with biventricular stimulation (male, 82 years) presenting with progressive dyspnea and chest pain 46 months after device implantation. A, adenosine stress CMR dynamic perfusion imaging (three slices in short-axis geometry) demonstrated inducible ischemia(white arrows) in the anterior/anteroseptal segments (apical and medial slice). B, wideband LGE (identical scan geometries) proved the absence of ischemic scar or myocardial fibrosis. C, corresponding invasive coronary angiography revealed eccentric high-grade stenosis of medial left anterior descending artery (black arrow). Approximate generator position is indicated by the asterisk

Overall, post–contrast cine imaging allowed for evaluation of 96.1 ± 6.1% of LV and 95.6 ± 6.6% of RV segments. For LGE imaging, LV and RV segmental evaluability amounted to 94.2 ± 7.8% and 95.8 ± 6.9%, respectively; T1- and T2 imaging resulted in 94.9 ± 7.2% and 91.3 ± 8.5% evaluable LV segments, respectively; perfusion imaging allowed for evaluation of 98.8 ± 2.2% of LV myocardial segments. In general, artifact occurrence was most common in the anterior LV segments; representative CMR imaging examples are provided in Figs. 1, 2, 3 and 4 and additional files 1–3 (video format: .mp4), respectively. Image quality of contrast-enhanced three-dimensional angiography of the pulmonary veins or thoracic aorta was unimpaired in all cases (100% evaluability).

**Fig. 5:**
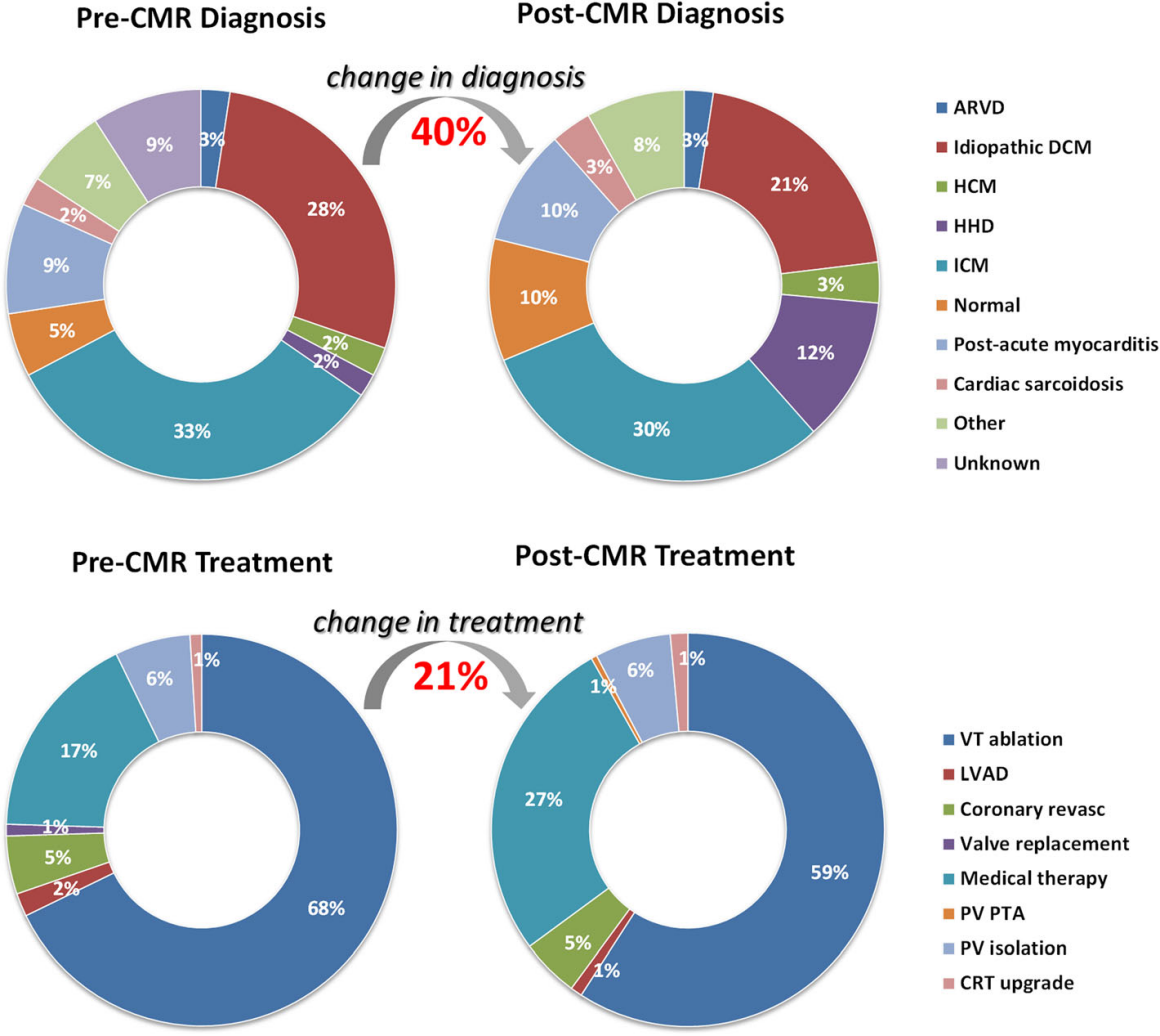
Doughnut chart to illustrate the relative distribution of cardiac disease categories and treatment decisions before (pre-CMR) and after CMR testing (post-CMR) resulting in the respective overall percentage change in diagnosis and treatment decisions (red numbers in bold). ARVC, arrhythmogenic right-ventricular cardiomyopathy; DCM, dilated cardiomyopathy; HCM, hypertrophic cardiomyopathy; HHD, hypertensive heart disease; ICM, ischemic cardiomyopathy; VT, ventricular tachycardia; LVAD, left ventricular assist device; Coronary revasc, coronary revascularization procedure; PV, pulmonary vein; PTA percutaneous transluminal angioplasty; CRT, cardiac resynchronization therapy

### CMR–based change in diagnosis

Overall, CMR imaging data resulted in a change in diagnosis in 40% (83/208) of ICD patients with a significant difference between primary versus secondary prevention ICD patients (37/115, 32% versus 46/93, 49%; *p* = 0.01; see Table 3 and Fig. 5); based on CMR imaging data, the percentages reclassified mainly affected the following diagnostic categories: hypertensive heart disease (22/83, 27%), post-acute myocarditis (13/83, 16%), dilated cardiomyopathy (13/83, 16%) and normal (10/83, 12%). In addition to mere reclassification of underlying cardiac disease, newly discovered and important diagnostic findings consisted of detection of LV thrombus (9/208, 4%) and identification of LGE-defined fibrosis in non-ischemic cardiomyopathy (75/208, 36%) representing ventricular substrate as potential target for ablation therapy. Finally, for the group comparison of “electrical instability” versus “worsening heart failure” no significant difference for “change in diagnosis” was found (59/156, 38% versus 24/52, 46%, respectively; *p* = 0.288).

**Table 3:** CMR-Based Change in Diagnosis

Post-CMR diagnosis	All patients (*n* = 208)	All patients Change in diagnosis	Primary prevention (*n* = 115)	Primary prevention Change in diagnosis	Secondary prevention (*n* = 93)	Secondary prevention Change in diagnosis
Acute MI	2	2 (1)	1	1 (1)	1	1 (1)
Cardiac storage disease	1	0 (0)	1	0 (0)	0	0 (0)
ARVC	5	3 (1)	1	0 (0)	4	3 (3)
Idiopathic DCM	43	13 (6)	33	9 (8)	10	4 (4)
HCM	7	2 (1)	3	1 (1)	4	1 (1)
HHD	25	22 (11)	11	11 (10)	14	11 (12)
ICM	63	8 (4)	40	2 (2)	23	6 (6)
NCCM	1	1 (0.5)	0	0 (0)	1	1 (1)
Normal (prior LGE (+) VT ablation lesions)	4	4 (2)	1	1 (1)	3	3 (3)
Normal	21	10 (5)	5	4 (3)	16	6 (6)
Post–acute myocarditis	20	13 (6)	9	5 (4)	11	8 (9)
PPCM	2	0 (0)	2	0 (0)	0	0 (0)
Cardiac sarcoidosis	7	4 (2)	4	2 (2)	3	2 (2)
Systemic sclerosis	1	0 (0)	0	0 (0)	1	0 (0)
TOF	1	0 (0)	0	0 (0)	1	0 (0)
VHD	5	1 (0.5)	4	1 (1)	1	0 (0)
Total	208	83 (40)	115	37 (32)	93	46 (49)*

Values are n (%) for all, primary and secondary prevention groups. *MI* myocardial infarction; *ARVC* arrhythmogenic right-ventricular cardiomyopathy; *DCM* dilative cardiomyopathy; *HCM* hypertrophic cardiomyopathy; *HHD* hypertensive heart disease; *ICM* ischemic cardiomyopathy; *NCCM* non-compaction cardiomyopathy; *LGE* late gadolinium enhancement; *VT* ventricular tachycardia; *PPCM* peripartum cardiomyopathy; *TOF* tetralogy of Fallot; *VHD* valvular heart disease. * *p* = 0.011 for the comparison of primary versus secondary prevention

### CMR–based change in treatment

The information gained from CMR imaging resulted in an overall change in treatment in 21% (43/208) with a similar distribution in primary versus secondary prevention ICD patients (25/115, 22% versus 18/93, 19%, *p* = 0.67; see Table 4 and Fig. 5). The main effect of the CMR results on treatment change can be gauged when focusing on patients referred for an invasive treatment: after CMR, a total of 123 of 141 patients initially scheduled for a VT ablation procedure subsequently underwent VT ablation while in the remaining 18 patients (13%) the treatment categories changed to medical therapy (*n* = 14) or coronary revascularization (*n* = 4). In more detail, the CMR findings giving rise to deferral of VT ablation were evidence of myocardial ischemia (n = 4), acute myocardial inflammation/cardiac sarcoidosis (n = 4), newly detected LV thrombus formation (*n* = 5), absence of CMR-identifiable structural heart disease and, thus, subsequent opportunity to initiate class IC antiarrhythmic drug therapy (*n* = 2), extreme LV wall thinning after transmural ischemic myocardial infarction (< 2 mm, *n* = 1), newly diagnosed arrhythmogenic RV dysplasia (end-stage disease with extensive RV fibrosis formation, *n* = 1) and in one patient the presence of CMR evidence of substantial pericardial fibrotic tissue formation/epicardial substrate after coronary artery bypass grafting who was deemed not suitable for an epicardial approach. Likewise, in patients initially considered for coronary revascularization (*n* = 14), the results of CMR imaging led to the following treatment strategies: coronary revascularization (*n* = 5), medical therapy (*n* = 8) and pulmonary venous isolation procedure (n = 1, negative adenosine stress CMR ruled out progression of known CAD and atrial fibrillation was deemed to represent the cause of progressive dyspnea).

**Table 4:** CMR-Based Change in Treatment

Post-CMR Treatment	All patients (n = 208)	All patients Change in treatment	Primary prevention (n = 115)	Primary prevention Change in treatment	Secondary prevention (n = 93)	Secondary prevention Change in treatment
Coronary Revasc	10	10 (5)	5	5 (4)	5	5 (5)
CRT upgrade	3	3 (1)	2	2 (2)	1	1 (1)
LVAD	2	1 (0.5)	2	1 (1)	0	0 (0)
Medical therapy	56	27 (13)	32	15 (13)	24	12 (13)
PVI	13	1 (0.5)	8	1 (1)	5	0 (0)
PV angioplasty	1	1 (0.5)	1	1 (1)	0	0 (0)
VT ablation	123	0 (0)	65	0 (0)	58	0 (0)
Total	208	43 (21)	115	25 (22)	93	18 (19)*

Values are n (%) for all, primary and secondary prevention groups. Coronary Revasc, coronary revascularization procedure; *CRT* cardiac resynchronization therapy *LVAD* left ventricular assist device; *PVI* pulmonary venous isolation procedure; *PV* pulmonary vein; *VT* ventricular tachycardia. * *p* = 0.673 for the comparison of primary versus secondary prevention

In addition, while *n* = 2 patients initially scheduled for device upgrade to CRT were not considered for this treatment after CMR detected extensive inferolateral infarction/scar tissue (thereby rendering CRT ineffective), another *n* = 3 patients were newly stratified for device upgrade based on accurate, CMR-derived volumetric determination of low systolic LV ejection fraction which had been rather overestimated by prior transthoracic echocardiography [12]. For the group comparison of “electrical instability” versus “worsening heart failure”, a statistically significant difference of “change in treatment” was noted (25/156, 16% versus 18/52, 35%, respectively; *p* = 0.004).

## Discussion

The main findings of the current CMR study in ICD patients presenting with electrical instability or worsening heart failure symptoms were as follows: (1) taking advantage of a standardized CMR protocol, a high proportion of diagnostic examinations (98%) with a consistently high ventricular segmental evaluability of cine and LGE imaging modules (94 to 96%) was achieved, (2) CMR testing resulted in a change in diagnosis in 40% of the overall population with a significant difference between primary and secondary prevention ICD patients (32% versus 49%, respectively), (3) CMR testing caused a change in treatment decisions in 21% of patients with no significant difference between primary and secondary prevention ICD patients (22 versus 19%, respectively).

There may be several reasons for the relatively high proportion of change in diagnosis particularly found in secondary prevention ICD patients: while in accordance with current recommendations, the common practice to perform echocardiography and invasive coronary angiography (i.e. assessment of ventricular dimensions/function and exclusion of significant CAD) as the sole diagnostic work-up prior to ICD implantation may not be sufficient to cover the whole range of myocardial tissue alterations possibly being detectable at the time of implantation. In particular, cardiac diseases mimicking LV hypertrophy (sarcoidosis, storage diseases etc.) may be misclassified as hypertensive heart disease in the presence of concomitant arterial hypertension and, thus, may go undetected (see Fig. 2, additional file 2). Notwithstanding, current guidelines regarding non-invasive diagnostic testing in survivors of sudden cardiac death defined the recommendation level for CMR imaging as “moderate” only (IIa C-EO, i.e. the diagnostic test may be useful/beneficial mainly based on clinical experience and/or expert opinion only) [13].

Study data in smaller populations suggested that the addition of CMR testing to the diagnostic work-up (i.e. “baseline” CMR imaging prior to ICD implantation) in patients with documented VT/survivors of sudden cardiac death yielded a high incremental diagnostic value with a resultant reassignment of about 50% of patients to a new or alternate diagnosis [14]. It must be noted, however, that baseline CMR imaging alone may not suffice to fully address the diagnostic dilemma: from a pathophysiological point of view, the cells of the intrinsic electrical conductance system are generally more susceptible to inflammation than myocardial cells [15, 16]. Hence, “indicator” arrhythmias (e.g. higher-grade AV-block, ventricular arrhythmias) may occur in earlier inflammatory disease stages even before any of the structural myocardial cell alterations which are detectable by CMR imaging (= inherent limit of detection of CMR imaging). However, for the time being ICD implantation constitutes the central component of treatment for patients with ischemic or non-ischemic cardiomyopathy, who are at risk for sudden cardiac death due to VT. Consequently, in consideration of the natural progression of myocardial diseases and/or fluctuating disease stages, diagnostic re-evaluation of ICD patients should be strongly considered, especially if life threatening arrhythmias or worsening heart failure symptoms reoccur over time. Previous research reported on the added value of CMR imaging in patients with MR–conditional pacemakers only which resulted in diagnostic or management-changing information in 63% of the patient cohort [17], while the present study data further corroborated the high impact of CMR-based information on clinical decision making in ICD patients.

The high diagnostic performance of CMR-based myocardial tissue characterization offers the unique ability to adequately monitor cardiac disease progression in the individual patient and in the current study, the information gain from CMR testing proved useful to re-route clinical pathways: clinical management/treatment decisions were changed in a high proportion of patients (21%). Most importantly, in the non-ischemic cardiomyopathy group LGE-defined ventricular substrate as a potential target for subsequent VT ablation was newly detected in 36% of ICD patients; since substrate-guided (“fibrosis-guided”) ablation has become a cornerstone for catheter treatment of complex ventricular arrhythmias such CMR-based information is of great clinical usefulness to determine overall suitability of the patient for ventricular ablation (i.e. expected complexity of the ablation procedure), the preferred route of access (i.e. endocardial or epicardial approach only or combined endo−/epicardial approach) and to facilitate a more targeted ablation with knowledge about the segmental location of potential substrate beforehand [18–20]. However, in the current study changes in access route/ablation strategy were not considered a CMR-based change in treatment but rather a readily available and complimentary information gain for the interventional electrophysiologist.

### Study limitations

While the study cohort closely reflected the clinical spectrum typically encountered at a highly specialized, tertiary care center, the data as such may only be applicable to a similar clinical scenario. Some referral bias may be present in the study population, since the decision to request CMR imaging has entirely been left to the referring electrophysiologist/cardiologist and critically-ill patients were generally not considered for a CMR study.

Importantly, while at our institution CMR imaging in device carriers is routinely carried out with a high degree of CMR imaging expertise (> 20 years of experience) following a previously established workflow taking advantage of more recent CMR sequence developments and with adherence to current guideline recommendations, local expertise and/or scanner availability may vary considerably and must be taken into account when implementing CMR imaging of ICD patients as an integral component of the diagnostic workup [5, 6, 21].

On a per patient level, a 2% rate of “non-diagnostic” studies was determined (i.e. studies for which there was no interpretable CMR imaging data) and this may be considered the total rate of non-usable study information based on the proposed CMR imaging protocol in ICD patients; in case CMR imaging was carried out successfully, the rates of non-diagnostic segmental evaluability of regional wall motion and wideband LGE were 4 and 6%, respectively.

## Conclusions

The present study data demonstrated the high impact of CMR imaging regarding diagnosis and clinical management in ICD patients presenting with electrical instability or worsening heart failure symptoms: CMR-based findings provided diagnostic and treatment changing information in a considerable proportion of ICD patients (40 and 21%, respectively). Hence, this work further contributed to the growing information base for CMR testing in its evolving role as an arbiter for profound clinical decision making and subsequent rerouting of clinical pathways in ICD patients either.

## Supplementary information

**Additional file 1.** CMR imaging in a single-chamber, secondary prevention ICD patient (male, 59 years; identical patient as shown in the still frame of Fig. 1) presenting with electrical storm (15 adequate episodes of antitachycardia pacing) 24.7 months after device implantation. Diagnostic change (+); management/treatment change (−): pre-CMR referral diagnosis: unknown cardiac disease; post-CMR diagnosis: post-infectious state of chronic myocarditis; pre-CMR treatment plan: ventricular tachycardia (VT) ablation; post-CMR treatment plan: targeted ventricular ablation procedure guided by LGE-defined ventricular substrate. A – D, post–contrast cine imaging in all cardiac standard geometries demonstrated regional wall motion abnormality (akinesis of basal inferolateral segment) with near-normal global systolic LV function (LVEF 52%); E – H, LGE CMR imaging in identical geometries revealed the presence of extensive, strictly epicardial regions of fibrosis. Approximate generator position is indicated by the asterisk.

**Additional file 2.** CMR imaging in a dual-chamber, secondary prevention ICD patient (male, 59 years; identical patient as shown in the still frame of Fig. 2) presenting with electrical storm (13 adequate shock episodes) 7.2 months after device implantation. Diagnostic change (+); management/treatment change (+): pre-CMR referral diagnosis: hypertensive heart disease; post-CMR diagnosis: acute disease state of cardiac sarcoidosis; pre-CMR treatment plan: VT ablation; post-CMR treatment plan: medical therapy including steroids. A – D, post–contrast cine imaging in all cardiac standard geometries showed concentric LV hypertrophy with mildly reduced global systolic LV function (LVEF 47%); E – H, LGE CMR imaging (identical geometries) identified extensive, mostly transmural areas of positive LGE in all septal and inferior LV segments with corresponding edema on T2-weighted turbo spin echo imaging (please refer to Fig. 2) consistent with an acute inflammatory disease state of cardiac sarcoidosis subsequently being confirmed by endomyocardial biopsy. Approximate generator position is indicated by the asterisk.

**Additional file 3.** CMR imaging in a dual-chamber, secondary prevention ICD patient (female, 58 years; identical patient as shown in the still frame of Fig. 3) presenting with worsening heart failure symptoms 27.9 months after device implantation. Diagnostic change (+); management/treatment change (−): pre-CMR referral diagnosis: suspected myocardial storage disease; post-CMR diagnosis: hypertrophic cardiomyopathy; pre-CMR treatment plan: medical therapy; post-CMR treatment plan: medical therapy. A – D, on post–contrast cine imaging (cardiac standard geometries) LV septal hypertrophy was identified with normal regional wall motion/normal global systolic LV function (LVEF 59%); E – H, LGE CMR imaging demarcated the typical pattern of multifocal islands/patchy fibrosis in the interventricular septum while the remaining LV myocardium was normal. Approximate generator position is indicated by the asterisk.

## Data Availability

Not applicable.

## Abbreviations

ATP: Antitachycardia pacing
CAD: Coronary artery disease
CMR: Cardiovascular magnetic resonance
CRT: Cardiac resynchronization therapy
ECG: Electrocardiogram
ICD: Implanted cardioverter defibrillator
LGE: Late-gadolinium enhancement
LV: Left ventricle/left ventricular
MR: Magnetic resonance
RV: Right ventricle/right ventricular
SAR: Specific absorption rate
SGE: Spoiled gradient echo
VT: Ventricular tachycardia

## References

[1] Russo RJ, Costa HS, Silva PD, Anderson JL, Arshad A, Biederman RW (2017). Assessing the risks associated with MRI in patients with a pacemaker or defibrillator. N Engl J Med.

[2] Nazarian S, Hansford R, Roguin A, Goldsher D, Zviman MM, Lardo AC (2011). A prospective evaluation of a protocol for magnetic resonance imaging of patients with implanted cardiac devices. Ann Intern Med.

[3] Nazarian S, Hansford R, Rahsepar AA, Weltin V, McVeigh D, Gucuk Ipek E (2017). Safety of magnetic resonance imaging in patients with cardiac devices. N Engl J Med.

[4] Nazarian S, Roguin A, Zviman MM, Lardo AC, Dickfeld TL, Calkins H (2006). Clinical utility and safety of a protocol for noncardiac and cardiac magnetic resonance imaging of patients with permanent pacemakers and implantable-cardioverter defibrillators at 1.5 tesla. Circulation.

[5] Hilbert S, Jahnke C, Loebe S, Oebel S, Weber A, Spampinato R (2018). Cardiovascular magnetic resonance imaging in patients with cardiac implantable electronic devices: a device-dependent imaging strategy for improved image quality. Eur Heart J Cardiovasc Imaging.

[6] Hilbert S, Weber A, Nehrke K, Bornert P, Schnackenburg B, Oebel S (2018). Artefact-free late gadolinium enhancement imaging in patients with implanted cardiac devices using a modified broadband sequence: current strategies and results from a real-world patient cohort. Europace.

[7] Schwitter J, Gold MR, Al Fagih A, Lee S, Peterson M, Ciuffo A, et al. Image quality of cardiac magnetic resonance imaging in patients with an implantable cardioverter defibrillator system designed for the magnetic resonance imaging environment. Circ Cardiovasc Imaging. 2016;9(5). 10.1161/CIRCIMAGING.115.004025 27151268

[8] Stevens SM, Tung R, Rashid S, Gima J, Cote S, Pavez G (2014). Device artifact reduction for magnetic resonance imaging of patients with implantable cardioverter-defibrillators and ventricular tachycardia: late gadolinium enhancement correlation with electroanatomic mapping. Heart Rhythm.

[9] Cerqueira MD, Weissman NJ, Dilsizian V, Jacobs AK, Kaul S, Laskey WK (2002). Standardized myocardial segmentation and nomenclature for tomographic imaging of the heart. A statement for healthcare professionals from the cardiac imaging Committee of the Council on clinical cardiology of the American Heart Association. Circulation.

[10] Rudski LG, Lai WW, Afilalo J, Hua L, Handschumacher MD, Chandrasekaran K (2010). Guidelines for the echocardiographic assessment of the right heart in adults: a report from the American Society of Echocardiography endorsed by the European Association of Echocardiography, a registered branch of the European Society of Cardiology, and the Canadian Society of Echocardiography. J Am Soc Echocardiogr.

[11] Almehmadi F, Joncas SX, Nevis I, Zahrani M, Bokhari M, Stirrat J (2014). Prevalence of myocardial fibrosis patterns in patients with systolic dysfunction: prognostic significance for the prediction of sudden cardiac arrest or appropriate implantable cardiac defibrillator therapy. Circ Cardiovasc Imaging.

[12] Bleeker GB, Kaandorp TA, Lamb HJ, Boersma E, Steendijk P, de Roos A (2006). Effect of posterolateral scar tissue on clinical and echocardiographic improvement after cardiac resynchronization therapy. Circulation.

[13] Al-Khatib SM, Stevenson WG, Ackerman MJ, Bryant WJ, Callans DJ, Curtis AB (2018). 2017 AHA/ACC/HRS guideline for Management of Patients with Ventricular Arrhythmias and the prevention of sudden cardiac death: a report of the American College of Cardiology/American Heart Association task force on clinical practice guidelines and the Heart Rhythm Society. J Am Coll Cardiol.

[14] White JA, Fine NM, Gula L, Yee R, Skanes A, Klein G (2012). Utility of cardiovascular magnetic resonance in identifying substrate for malignant ventricular arrhythmias. Circ Cardiovasc Imaging.

[15] Lazzerini PE, Capecchi PL, El-Sherif N, Laghi-Pasini F, Boutjdir M (2018). Emerging arrhythmic risk of autoimmune and inflammatory cardiac channelopathies. J Am Heart Assoc.

[16] Rosenthal N (2017). A Guardian of the heartbeat. N Engl J Med.

[17] Raphael CE, Vassiliou V, Alpendurada F, Prasad SK, Pennell DJ, Mohiaddin RH (2016). Clinical value of cardiovascular magnetic resonance in patients with MR-conditional pacemakers. Eur Heart J Cardiovasc Imaging.

[18] Acosta J, Fernandez-Armenta J, Penela D, Andreu D, Borras R, Vassanelli F (2016). Infarct transmurality as a criterion for first-line endo-epicardial substrate-guided ventricular tachycardia ablation in ischemic cardiomyopathy. Heart Rhythm.

[19] Andreu D, Ortiz-Perez JT, Boussy T, Fernandez-Armenta J, de Caralt TM, Perea RJ (2014). Usefulness of contrast-enhanced cardiac magnetic resonance in identifying the ventricular arrhythmia substrate and the approach needed for ablation. Eur Heart J.

[20] Oebel S, Dinov B, Arya A, Hilbert S, Sommer P, Bollmann A (2017). ECG morphology of premature ventricular contractions predicts the presence of myocardial fibrotic substrate on cardiac magnetic resonance imaging in patients undergoing ablation. J Cardiovasc Electrophysiol.

[21] Indik JH, Gimbel JR, Abe H, Alkmim-Teixeira R, Birgersdotter-Green U, Clarke GD (2017). 2017 HRS expert consensus statement on magnetic resonance imaging and radiation exposure in patients with cardiovascular implantable electronic devices. Heart Rhythm.

